# Data Clustering Using Moth-Flame Optimization Algorithm

**DOI:** 10.3390/s21124086

**Published:** 2021-06-14

**Authors:** Tribhuvan Singh, Nitin Saxena, Manju Khurana, Dilbag Singh, Mohamed Abdalla, Hammam Alshazly

**Affiliations:** 1 Department of Computer Science and Engineering, Siksha ‘O’ Anusandhan (Deemed to be University), Bhubaneswar, Odisha 751030, India; tribhuvansingh@soa.ac.in; 2 Department of Computer Science and Engineering, Thapar Institute of Engineering and Technology, Patiala, Punjab 147004, India; nitin.saxena@thapar.edu (N.S.); manju.khurana@thapar.edu (M.K.); 3 School of Engineering and Applied Sciences, Bennett University, Greater Noida 201310, India; dggill2@gmail.com; 4 Department of Mathematics, Faculty of Science, King Khalid University, Abha 62529, Saudi Arabia; moabdalla@kku.edu.sa; 5 Department of Mathematics, Faculty of Science, South Valley University, Qena 83523, Egypt; 6 Faculty of Computers and Information, South Valley University, Qena 83523, Egypt

**Keywords:** data clustering, data mining, k-means, moth flame optimization, meta-heuristic

## Abstract

A k-means algorithm is a method for clustering that has already gained a wide range of acceptability. However, its performance extremely depends on the opening cluster centers. Besides, due to weak exploration capability, it is easily stuck at local optima. Recently, a new metaheuristic called Moth Flame Optimizer (MFO) is proposed to handle complex problems. MFO simulates the moths intelligence, known as transverse orientation, used to navigate in nature. In various research work, the performance of MFO is found quite satisfactory. This paper suggests a novel heuristic approach based on the MFO to solve data clustering problems. To validate the competitiveness of the proposed approach, various experiments have been conducted using Shape and UCI benchmark datasets. The proposed approach is compared with five state-of-art algorithms over twelve datasets. The mean performance of the proposed algorithm is superior on 10 datasets and comparable in remaining two datasets. The analysis of experimental results confirms the efficacy of the suggested approach.

## 1. Introduction

Data clustering methods are being widely implemented in various real-world applications such as data mining [1], machine learning [2], information retrieval [3,4], pattern recognition [5,6,7], face clustering and recognition [8,9,10], wireless sensor networks [11], etc. The objective of this method is to partition data objects in such a way to minimize accumulated distances between data objects and their respective centroids. After clustering, the data objects in a cluster should be as similar as possible with each other and should be vastly different from the items of other clusters. A number of algorithms have been proposed to solve the data clustering problem. K-means is one among the popular algorithms to handle clustering problem. K-means algorithm is simple and efficient but the accuracy of its result highly dependent on initially selected cluster centers, hence prone to trap in local optima solution. Data clustering is one of the NP-hard problems and hence difficult to solve using deterministic algorithms. Being an NP-hard problem, deterministic approaches cause local entrapment which in turn, affects the overall performance of the algorithm.

Although the approach adopted by the k-means is being widely accepted for dealing with the clustering problems, it is deterministic in nature except for initialization. Therefore, to deal with data clustering problems a better approach could be expected compared to the k-means algorithm. Alternatively, the heuristic clustering algorithms are being widely used these days as a substitute for the conventional clustering technique. These heuristic clustering algorithms target their goals with the random search in the specified search domain. These algorithms are unable to capture the exact values, but, they try to find the values that are as close as possible to the exact solution. In this view, it is considered as an optimization problem in which the sum of the intra-cluster distances between the cluster center and associated data objects need to be minimized. To solve this problem, nature-inspired algorithms are being widely used these days [12,13,14,15,16] along with [17,18]. The problem of data clustering has attracted the attention of many researchers from different domains of science and engineering and it is still an important area of research in data mining.

Due to the involvement of non-linear objective function and large search domain in data clustering problems, the algorithms find difficulty during the optimization process. In such a case, the selection of an appropriate optimization algorithm for solving this problem becomes more important. An optimization algorithm for solving data clustering problems needs to have a balanced and proper mechanism of exploitation and exploration. Hatamlou proposes a black hole algorithm guided approach for data clustering [19]. A cuckoo search optimization algorithm based approach for data clustering is presented in [20]. A detailed review of data clustering using particle swarm optimization and its variants have been presented in [21]. Han et al. [22] proposed a modified gravitational search algorithm (GSA) for data clustering. They have included a new mechanism in GSA for improving the global search ability of solutions during the optimization. Apart from this, the use of chaotic sequences instead of random numbers improves the overall performance of optimization algorithms [23,24]. Inspired by the chaos theory, Chuang et al. [25] proposed a method for data clustering using chaotic map and PSO. Additionally, another approach based on the chaotic sequences is presented in [26] for data clustering. Another approach for data clustering based on chaotic sequence harris hawks optimizer is proposed in [27]. A chaotic number and opposition learning based method for data clustering is presented in [28]. A levy flight-based cuckoo search algorithm for data clustering is proposed in [29]. Abdulwahab et al. [30] suggested a levy flight guided approach for data clustering. The opposition learning-based approach is being successfully implemented in optimization algorithms to improve diversity during the search [31]. Kumar et al. [32] proposed an opposition learning and Cauchy operator-based scheme for data clustering. Sun et al. [33] introduced an opposition learning and random local perturbation guided monarch butterfly optimization algorithm for data clustering. Nasiri et al. [34] suggested a whale optimization algorithm based approach to handle data clustering problems. The authors have validated the effectiveness of their approach based on various comparative performance analysis. A magnetic optimization algorithm-guided approach is presented for data clustering in [14]. A hybrid approach based on exponential Grey Wolf Optimizer (GWO) and Whale Optimization Algorithm (WOA) is presented in [35] for dealing with data clustering problem. A variance-based differential evolution approach for data clustering is suggested in [36]. Zhou et al. [37] proposed a symbiotic organism search algorithm-guided approach to handle data clustering problems. A cuttlefish optimization algorithm based-approach for data clustering is presented in [38]. A density-based clusterability measure is proposed to measure the prominence of the clustering structure in the time-series data [39]. A whale optimization algorithm based approach for data clustering is presented in [40].

Although various optimization algorithms have already been suggested, none of them can outperform all other algorithms in all benchmark datasets of data clustering. A general idea of no free lunch theorem [41] states that not a single algorithm can defeat all other algorithms in all test problems. This could be the reason behind the development of various optimization algorithms for data clustering. The methods of exploitation and exploration are two main aspects that need to be balanced to achieve the desired goal in reasonable time. However, these two aspects are conflicting in nature. Excessive exploitation causes premature convergence; while excessive exploration causes random search. A proper exploitation method intentionally tries to search in the neighborhood of good solutions found so far. However, exploration is supposed to capture good solutions from the different regions of a search domain. Therefore, an optimization algorithm designed to solve the data clustering problem needs to have the capability of searching the solutions around the neighborhood of good solutions and also capturing those solutions that are far away from the poor solutions. In order to design an optimization algorithm that achieves the aforementioned capability, the programmer has to concentrate on how to update the position vectors of the solutions in an efficient manner.

Recently, Moth Flame Optimization (MFO) [42] for solving global optimization problems and real-world applications is proposed. The effectiveness of MFO in terms of population diversity and convergence rate has already been justified while solving multifaceted problems. A new method based on the MFO for data clustering is proposed in this paper. The competitiveness of the MFO such as fast convergence towards the global optimal solution and ability to avoid local optima is utilized in this study. Our main objective is to divide the data objects into clusters using MFO with better accuracy and complete coverage of search space to the existing methods. In short, the novelty and contribution of this paper are highlighted below.

- MFO based approach for data clustering is presented.
- The proposed approach is evaluated using 12 machine learning benchmark datasets.
- The quality of the solutions produced by the proposed approach is compared against five well-known algorithms.
- Three statistical tests have been performed to measure the quality of the proposed approach statistically.
- Based on experimental values, statistical values, and convergence curves, the efficacy of the proposed approach is justified.

The rest of the paper is structured as follows. In Section 2, the fundamental concepts of data clustering and the MFO algorithm is illustrated. Section 3 explains the proposed approach in detail. In Section 4, the benchmark datasets and experimental setup are described. The result analysis and discussion are given in Section 5 and Section 6. Section 7 highlights the conclusions and future research directions.

## 2. Basic Concepts

### 2.1. Clustering

Data clustering is a method to group the given set of *N* data objects into *K* clusters. The primary objective of clustering is that the data objects belong to the same cluster must be highly similar, whereas the data objects must show high dissimilarity to those belonging to different clusters [43,44]. The similarity and dissimilarity are measured using Euclidean distance. Precisely, the sum of the square of Euclidean distances between each data object and its associated cluster center is calculated. Hence, data clustering intents to minimize the sum of intra-cluster distances. Formally it can be defined as follows:

Let dataset $O={\left\{O_{1},O_{2},\cdots O_{N}\right\}}^{T}$ contains *N* data objects each with *D* attributes (dimensions) i.e., $O_{i}=\left\{o_{i}^{1},o_{i}^{2},\cdots o_{i}^{D}\right\}$, for *i* = 1 to *N*. The scalar value $o_{i}^{d}$ denotes the $d^{th}$ attribute of $i^{th}$ data object $O_{i}$. In this way, the whole dataset forms a matrix of N×D order such as

(1) $$O=\left[\begin{array}{c} O_{1}\\ \vdots \\ O_{N} \end{array}\right]=\left[\begin{array}{cccc} o_{1}^{1} & o_{1}^{2} & \cdots & o_{1}^{D}\\ \vdots & \vdots & \vdots & \vdots \\ o_{N}^{1} & o_{N}^{2} & \cdots & o_{N}^{D} \end{array}\right]$$

At the end of clustering process a set *Z* of *K* cluster formed as $Z=Z_{1},Z_{2},\ldots Z_{K}$. Each object belongs to exactly one of the formed K clusters such that objects sharing the same cluster are most similar, while objects belonging to separate clusters should be sufficiently distinct. The produced clusters and dataset should also adhere to the following criterion:

1. $Z_{k}\neq \emptyset \;\forall k=1\;to\;K$
2. $Z_{u}\cap Z_{v}=\emptyset$*u*, *v* = 1 …*K*; u≠v
3. $\overset{K}{\underset{k=1}{⋃}}Z_{k}=O$

In order to identify clusters and their most suitable data objects, Equation (2) is used as a fitness function in this paper to achieve the above objectives.

(2) $$F\left(O,Z\right)=\sum_{i=1}^{N}\sum_{j=1}^{K}w_{ij}\left|\right|\left(O_{i}-Z_{j}\right){\left|\right|}^{2}$$

where the fitness value *F(O, Z)* is to be minimized. The Euclidean distance between a $i^{th}$ data object, $O_{i}$ and the $j^{th}$ cluster center, $Z_{j}$ is ||($O_{i}-Z_{j}$)||. $w_{ij}$ is the weight associated with data object $O_{i}$ and cluster *j*. The value of $w_{ij}$ is assigned to 1 if $i^{th}$ data object is with $j^{th}$ cluster, else $w_{ij}$ = 0.

### 2.2. Moth Flame Optimization (MFO)

The MFO [42] is a swarm-based optimization algorithm to solve global optimization problems. This algorithm depicts moths intelligence used to navigate in nature which is known as transverse orientation. The algorithm assumes moths and their positions to be candidate solutions and problem’s variables, respectively. Moths can fly in multidimensional search domains with changing their position vectors. Like other optimization algorithms, MFO initializes candidate solutions randomly within the boundary range as follows.

(3) $$S_{p}=\hat{l}+\beta .\left(\hat{u}-\hat{l}\right)$$

where $S_{p}\left(1\leq p\leq P\right)$ is the $p^{th}$ solution and *P* is the population size. β is a random number in (0, 1). $\hat{l}$ and $\hat{u}$ represent the lower and upper bounds for the problem taken under consideration and . is a point-by-point multiplication. The population i.e., set of all solutions are represented in P×D size matrix form as follows.

(4) $$M=\left[\begin{array}{ccccc} S_{1,1} & S_{1,2} & \cdots & \cdots & S_{1,D}\\ S_{2,1} & S_{2,2} & \cdots & \cdots & S_{2,D}\\ \vdots & \vdots & \vdots & \vdots & \vdots \\ S_{P,1} & S_{P,2} & \cdots & \cdots & S_{P,D} \end{array}\right]$$

where *D* the number of variables, represent the dimension of the problem taken under consideration. After random initialization, each solution is evaluated using fitness function and stored in a matrix of size P×1 as follows.

(5) $$FM=\left[\begin{array}{c} FS_{1}\\ FS_{2}\\ \vdots \\ FS_{P} \end{array}\right]$$

where $FS_{p}$ is the fitness value of $p^{th}$ solution. Another component in MFO algorithm is flames. Similar to the moths, flames are also matrix of size P×D and represented as follows.

(6) $$F=\left[\begin{array}{ccccc} F_{1,1} & F_{1,2} & \cdots & \cdots & F_{1,D}\\ F_{2,1} & F_{2,2} & \cdots & \cdots & F_{2,D}\\ \vdots & \vdots & \vdots & \vdots & \vdots \\ F_{P,1} & F_{P,2} & \cdots & \cdots & F_{P,D} \end{array}\right]$$

The fitness of each flame is evaluated and stored in a matrix of size P×1 as follows.

(7) $$FF=\left[\begin{array}{c} FF_{1}\\ FF_{2}\\ \vdots \\ FF_{P} \end{array}\right]$$

Here, it is important to note that both moths and flames are solutions. The difference is, moths represent the candidate solutions and work as search agents to capture the optimal solution. On the other hand, flame keeps the top solutions searched so far and ensures that the best solution is never lost.

Moths progress in search space is controlled through logarithmic spiral function, which simulates moth’s transverse orientation navigation. The positions of the $p^{th}$ moth $S_{p}$ is updated using following function:

(8) $$L\left(S_{p},F_{q}\right)=D_{p}.e^{bt}.cos\left(2\pi t\right)+F_{q}$$

where $D_{p}$ represents the distance between the $p^{th}$ moth and $q^{th}$ flame, *t* is a random number that varies from −1 to 1, and *b* is a constant used to define the shape of the logarithmic spiral. Here, $D_{p}$ is calculated as follows.

(9) $$D_{p}=\left|F_{q}-S_{p}\right|$$

where $S_{p}$ and $F_{q}$ are the $p^{th}$ moth and $q^{th}$ flame, respectively. The process continues until the termination condition is met. In the next Section, MFO guided approach for data clustering is presented.

## 3. Moth Flame Optimization for Data Clustering

The Moth-flame optimization algorithm is one of the nature-inspired algorithms to solve optimization problems. Since data clustering is one of the NP-hard problems, MFO is a viable candidate to solve it. MFO is a stochastic algorithm and can produce an optimal or near-optimal solution efficiently. It is also designed in such a way that it avoids local optima and converges towards a globally optimal solution. This motivates us to employ MFO for identifying clusters of data objects in the given datasets.

As mentioned in definition of clustering problem, the number of clusters *K* is predefined, finally represented as *Z* = $Z_{1},Z_{2},\ldots Z_{K}$. Each cluster $Z_{k}$, *k* = 1 to *K*, preserves its centre. In order to find optimal solution of clustering problem using MFO algorithm, each moth corresponds to *K* cluster centres. Let *M* = $\{{S_{1},S_{2},\cdots S_{P})\}}^{T}$ is the set of *P* moths, the $p^{th}$ moth $S_{p}$ is represented as

(10) $$S_{p}=\left[\begin{array}{c} S_{p1}\\ S_{p2}\\ \vdots \\ S_{pK} \end{array}\right]=\left[\begin{array}{cccc} s_{p1}^{1} & s_{p1}^{2} & \cdots & s_{p1}^{D}\\ s_{p2}^{1} & s_{p2}^{2} & \cdots & s_{p2}^{D}\\ \vdots & \vdots & \vdots & \vdots \\ s_{pK}^{1} & s_{pK}^{2} & \cdots & s_{pK}^{D} \end{array}\right]$$

where, $S_{pk}$ is the centre of $k^{th}$ cluster in the $p^{th}$ moth. Precisely, $Z_{pk}=X_{i}$, ∀*k* = 1 to *K*, i.e., $\left[s_{pk}^{1},s_{pk}^{2},\ldots s_{pk}^{D}\right]=\left[x_{i}^{1},x_{i}^{2},\ldots x_{i}^{D}\right]$, ∃! i ∈ [1, P], a unique data object of the dimension *D* from the dataset. In general, the set of moths is represented in the form of matrix of the order (PK×D) as:

(11) $$M=\left[\begin{array}{c} S_{1}\\ \vdots \\ S_{p}\\ \vdots \\ S_{P} \end{array}\right]=\left[\begin{array}{c} S_{11}\\ \vdots \\ S_{1K}\\ \vdots \\ S_{p1}\\ \vdots \\ S_{pK}\\ \vdots \\ S_{P1}\\ \vdots \\ S_{PK} \end{array}\right]=\left[\begin{array}{cccc} s_{11}^{1} & s_{11}^{2} & \cdots & s_{11}^{D}\\ \vdots & \vdots & \vdots & \vdots \\ s_{1K}^{1} & s_{1K}^{2} & \cdots & s_{1K}^{D}\\ \vdots & \vdots & \vdots & \vdots \\ \vdots & \vdots & \vdots & \vdots \\ s_{p1}^{1} & s_{p1}^{2} & \cdots & s_{p1}^{D}\\ \vdots & \vdots & \vdots & \vdots \\ s_{pK}^{1} & s_{pK}^{2} & \cdots & s_{pK}^{D}\\ \vdots & \vdots & \vdots & \vdots \\ \vdots & \vdots & \vdots & \vdots \\ s_{P1}^{1} & s_{P1}^{2} & \cdots & s_{P1}^{D}\\ \vdots & \vdots & \vdots & \vdots \\ s_{pK}^{1} & s_{pK}^{2} & \cdots & s_{pK}^{D} \end{array}\right]$$

The proposed approach models the clustering as a minimization problem where intra-cluster distance work as objective function as mentioned in (2). The fitness of each moth is computed and stored in PK×1 order column vector FM.

(12) $$FM=\left[\begin{array}{c} FS_{1}\\ \vdots \\ FS_{p}\\ \vdots \\ FS_{P} \end{array}\right]=\left[\begin{array}{c} FS_{11}\\ \vdots \\ FS_{1K}\\ \vdots \\ FS_{p1}\\ \vdots \\ FS_{pK}\\ \vdots \\ FS_{P1}\\ \vdots \\ FS_{PK} \end{array}\right]$$

Flames $F={\left\{F_{1},\ldots F_{q}\ldots F_{P}\right\}}^{T}$ and corresponding fitness $FF={\left\{FF_{1},\ldots FF_{q}\ldots FF_{P}\right\}}^{T}$ matrices are populated with sorted moths positions and their respective fitness in ascending order with respect to fitness. Here ∀q, *q* = 1 to *P*, $F_{q}=S_{t}$ such that $S_{t}\in M$ and $<FF_{1},FF_{2}\cdots FF_{P}>$ are sorted in ascending order.

Moths iterative update their positions according to logarithmic spiral function defined in Equation (8) in turn update flames to retain top P solutions in ascending order until termination condition met.

### 3.1. The Procedure

The following steps are derived from the above discussion for the proposed MFO based clustering algorithm. It is assumed that dataset *X* of dimension *D* with *N* data objects is in place and available for processing. The number of clusters *K* is also initialized with a suitable integer.

Step1 **Initialization**: Populate the position of moths *M* randomly with *P* candidate solutions, i.e., *M* = ${\left\{S_{1},S_{2},\ldots M_{P}\right\}}^{T}$. Each candidate solution $S_{p}$ includes *K* centres of dimension *D*.
Step2 **Moths Fitness Computation:** Compute the fitness value of each moth initialized in step 1 using (2) and store it in a column vector FM = ${\left\{FM_{1},FM_{2},\ldots FM_{P}\right\}}^{T}$.
Step3 **Flames Generation:** Store the moths fitness values column vector FM in sorted form in flames fitness column vector FF = ${\left\{FF_{1},FF_{2},\ldots FF_{P}\right\}}^{T}$. Generate the flames $F={\left\{F_{1},F_{2},\ldots F_{P})\right\}}^{T}$, by placing the individual moth corresponding to their fitness value in FF respectively.
Step4 **Update Moths Position:** Each $p^{th}$ Moth’s positions $S_{p}$ is updated using the $p^{th}$ flame $F_{p}$ and logarithmic spiral function.
Step5 **Update Flames:** Flames and their corresponding fitness are updated by taking top *P* positions from previous flames and updated moth position.
Step6 **Test Termination Condition:** If the termination condition is satisfied, the algorithm terminates. Otherwise, go to Step 4 for the next iteration.

The proposed MFO based clustering algorithm is summarized in Algorithm 1.
**Algorithm 1** MFO based Clustering Algorithm.**Input:**(i)O = D dimension dataset with N data objects(ii)K = Number of clusters**Output:**(i)Z = Set of K clusters $\left\{Z_{1},Z_{2}\ldots Z_{K}\right\}$ of data objects**Begin**1:InitializeP = population sizeMaxIter = maximum number of iterations$\forall_{p=1}^{P}S_{p}$ = Each Moth with K random cluster centre2:itr = 13:**while** itr<MaxItr **do**4:    **for** p = 1 to P **do**5:        **for** i = 1 to N **do**6:Calculate the Euclidean distance of each data object $O_{i}$ to cluster centres of $S_{p}$7:Assign $O_{i}$ to nearest cluster centres of $S_{pk}$, k= 1 to K8:Calculate the fitness using (2)9:        **end for**10:    **end for**11:Populate Flames F and their fitness FF12:Update Moths positions using Flames and logarithmic spiral function mentioned in 813:**end while**14:Assign Z = $F_{1}$15:return Z**End**

### 3.2. Analysis of Time Complexity

In this subsection, time complexity and space complexity are presented in terms of big-oh notation. In this work, the common parameters for all approaches are the number of independent runs *R*, population size *P*, and the maximum number of iterations *Maxitr*. For the proposed approach, a careful observation indicates that the time complexity will be O(Maxitr×P×N). Here, *N* is the number of data objects in a benchmark dataset taken under consideration. Hence this number varies with respect to the dataset. Therefore, based on common parameters, the time complexity of the proposed approach is O(Maxitr×P). On the other hand, space complexity is the total amount of memory space used by the benchmark dataset and other variables during the program execution. Simple variables such as *P*, *Maxitr* etc. need constant space as the size of these data types is constant. Therefore, simple variables add a space complexity of O(1). However, to store the randomly initialized and intermediate populations in the memory, it needs O(P×K×D). Here, *K* is the number of clusters present in a benchmark dataset, whereas *D* is the number of features in a benchmark dataset. *P* is the population size.

## 4. Experimental Setup

The quality of the solutions of the proposed algorithm was compared against Black Hole Algorithm (BHA) [19], Multi-Verse Optimizer (MVO) [45], Harris Hawks Optimizer (HHO) [46], Grey Wolf Optimizer (GWO) [47], and K-means algorithm [44]. The parameters of BHA, MVO, HHO, GWO, and k-means algorithms were set according to their corresponding references [19,44,45,46,47], respectively. In this study, three parameters were common for all the approaches. The values of these common parameters were considered as follows:

- Population size = 50
- Maximum iterations = 1000
- Independent runs = 20

The details of benchmark datasets used in this study are given in Table 1 and Table 2. All the employed datasets used multivariate real valued features for characterizing individual objects.

## 5. Results Analysis

The experimental results of algorithms for benchmark datasets are given in Table 3 and Table 4. These tables represent the quantitative values described with Equation (2). The statistical metrics best, worst, mean and standard deviation of achieved objective values by participating algorithms are compared. Since the performance of metaheuristic randomized algorithms are not deterministic, best and worst values in different runs may reasonably represent upper and lower performance bounds respectively. The mean may represent the center of all objective function values obtained in multiple runs. In addition to that standard deviation helps to understand the performance consistency of algorithm. Lower the standard deviation, more consistent the performance of algorithm around mean. Table 5 represents the relative average rank of the algorithms considered in this study. This table indicates that the proposed approach outperformed all other approaches considered in this study. Whereas, the k-means algorithm observed the worst performance in all benchmark datasets.

Iman-Davenport [48], Friedman [49], and Holm [50] tests were carried out to demonstrate the effectiveness of the algorithms in solving the data clustering problem. Table 6 and Table 7 show the experimental values of Iman-Davenport and Friedman tests for the Shape and UCI datasets, respectively. The rejection of the null hypothesis for both cases confirmed that there was a significant difference in the performances of the algorithms. Therefore, a post hoc test (Holm test) was performed to show the efficacy of the best performing algorithm against the rest of the algorithms. Table 8 and Table 9 represent the experimental results of the Holm test for Shape and UCI datasets, respectively. The rejection of the null hypothesis for k-means and MVO algorithms indicated that the proposed approach was statistically better than these algorithms. The null hypothesis was not rejected for GWO, HHO, and BHA which indicated that the performance of these algorithms was comparable with the proposed approach. However, Table 5 shows that the proposed approach was better than GWO, HHO, and BHA.

**Figure 1:**
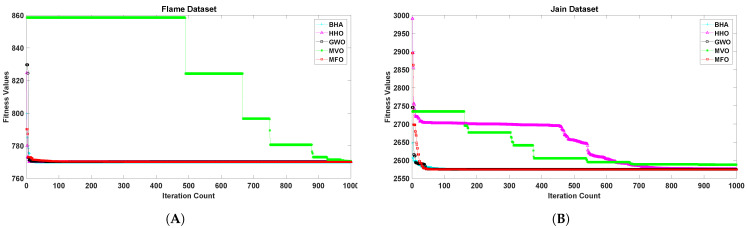
Variation in the best fitness values of algorithms for datasets (**A**): Flame, (**B**): Jain with respect to iterations.

**Figure 2:**
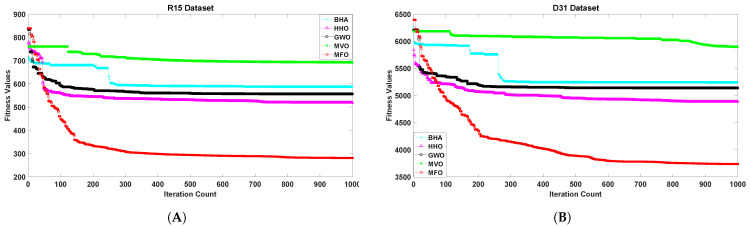
Variation in the best fitness values of algorithms for datasets (**A**): R15, (**B**): D31 with respect to iterations.

**Figure 3:**
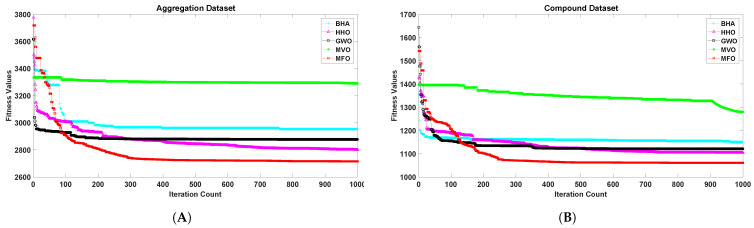
Variation in the best fitness values of algorithms for datasets (**A**): Aggregation, (**B**): Compound with respect to iterations.

**Figure 4:**
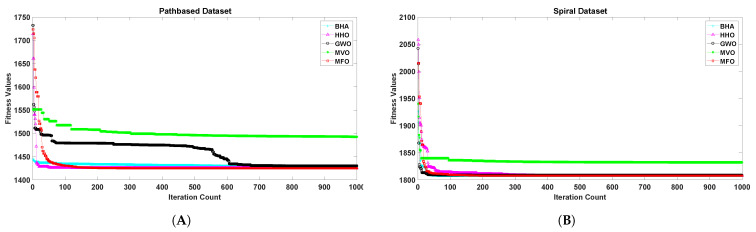
Variation in the best fitness values of algorithms for datasets (**A**): Pathbased, (**B**): Spiral with respect to iterations.

**Figure 5:**
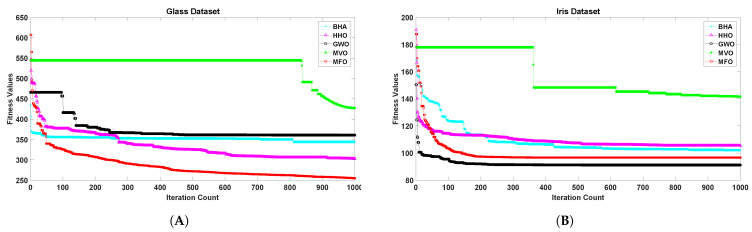
Variation in the best fitness values of algorithms for datasets (**A**): Glass, (**B**): Iris with respect to iterations.

**Figure 6:**
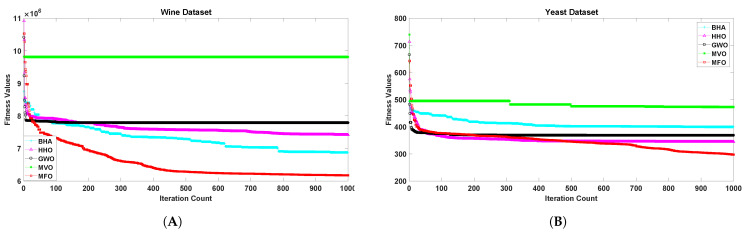
Variation in the best fitness values of algorithms for datasets (**A**): Wine, (**B**): Yeast with respect to iterations.

Figure 1, Figure 2, Figure 3, Figure 4, Figure 5 and Figure 6 show the convergence curves of algorithms for benchmark datasets taken under consideration. These curves show that the convergence rate of the suggested approach was much better than other approaches. Comparing algorithms using the rate of convergence allowed us to analyze how quick they captured the optimal solution.

## 6. Discussion

To further strengthen the results mentioned in the previous section, Table 10, Table 11, Table 12, Table 13, Table 14, Table 15, Table 16, Table 17, Table 18, Table 19, Table 20 and Table 21 represent the best centroids for D31, R15, Jain, Flame, Aggregation, Compound, Pathbased, Spiral, Glass, Iris, Wine, and Yeast datasets, respectively obtained by the suggested approach. In these tables, C1,C2,⋯,CK represent the best centroids obtained by the proposed approach, whereas F1,F2,⋯,FD represent the feature/attribute number of the dataset taken under consideration. Based on these centroids, the best value of the sum of fitness values of the respective benchmark dataset in Table 3 and Table 4 can be validated. By assigning each data object of a benchmark dataset to the respective centroid in Table 10, Table 11, Table 12, Table 13, Table 14, Table 15, Table 16, Table 17, Table 18, Table 19, Table 20 and Table 21, the best values in Table 3 and Table 4 are ideally expected. This can be understood as, by putting all the 240 data objects of the Flame dataset to one of the centroids between the two centroids that are presented in Table 13, the best value of the fitness function found by the MFO algorithm for the Flame dataset should be 770.09978. If this value (770.09978) is not found, then either the best value (770.09978) reported in Table 3 or the best centroids reported in Table 13 or both allocations are wrong. This method can be applied to validate the best value of the remaining benchmark datasets.

## 7. Conclusions and Future Research Directions

Various optimization algorithms based on natural phenomena have been used to solve complex problems. Moth flame optimization algorithm is one of them that uses the navigation behavior of moths at night. In this paper, a moth flame optimizer guided approach is suggested for data clustering. The effectiveness of the suggested method is validated using twelve standard benchmark datasets. Various comparisons using the experimental values have proved the effectiveness and competitiveness of the suggested method. The proposed approach achieves better accuracy and complete coverage of search space in comparison to the existing methods.

In the future, research can be carried out to solve some other real-world problems of clustering such as optimal controller selection in wireless sensor networks, controller placement problems in a software-defined network, clustering in image segmentation by using the proposed approach. The suggested approach can also be extended to solve multiobjective optimization problems.

## Figures and Tables

**Table 1:** Shape datasets.

Name	#Instances	#Features	#Classes	Year of Publication	Constructor	Dataset Objective
Flame	240	2	2	2007	L. Fu and E. Medico	DNA microarray data
Jain	373	2	2	2005	A. Jain and M. Law	Consensus function
R15	600	2	15	2002	C.J. Veenman et al.	Maximum variance clustering
D31	3100	2	31	2002	C.J. Veenman et al.	Maximum variance clustering
Aggregation	788	2	7	2007	A. Gionis et al.	Aggregating set of clusterings into single one
Compound	399	2	6	1971	C.T. Zahn	Detecting and describing gestalt clusters
Pathbased	300	2	3	2008	H. Chang and D.Y. Yeung	Robust path-based spectral clustering
Spiral	312	2	3	2008	H. Chang and D.Y. Yeung	Robust path-based spectral clustering

**Table 2:** UCI datasets.

Name	#Instances	#Features	#Classes	Year of Publication	Constructor	Dataset Objective
Iris	150	4	3	1936	R.A. Fisher	To predict class of iris plant
Glass	214	9	7	1987	B. German	To define the glass in terms of their oxide content
Yeast	1484	8	10	1991	Kenta Nakai	Predicting the cellular localization sites of proteins
Wine	178	13	3	1988	M. Forina et al.	Using chemical analysis to determine the origin of wines

**Table 3:** Comparison of objective values of MFO, BHA, MVO, HHO, GWO, k-means algorithms.

Dataset	Criteria	MFO	BHA	MVO	HHO	GWO	K-Means
	Best	770.09978	769.9661518	770.4754577	769.9927543	770.132897	778.2235737
	Worst	790.112976	799.8706082	883.7379944	881.5972455	862.9405109	882.2962778
Flame	Mean	770.312682	770.0151324	820.6166847	773.1715904	774.9724323	825.0039174
	Std	0.934345	0.048796151	25.9021804	2.197184147	3.456581526	32.9362996
	Best	2574.2421	2574.241619	2587.729382	2574.24163	2574.596821	2649.716145
	Worst	2895.455517	2872.057675	3317.743133	3243.435326	3351.013971	3348.696543
Jain	Mean	2578.583781	2575.625939	2783.852076	2609.24115	2604.748372	2898.773998
	Std	5.434534	1.216412722	152.227768	50.99513097	46.7066178	190.2690739
	Best	281.130101	587.7144266	692.2279482	518.9798792	555.9717927	766.9066841
	Worst	838.491757	882.9244343	914.6624615	912.7932725	933.2892028	901.9060829
R15	Mean	334.6612324	686.732183	830.124701	680.9312354	676.6880723	839.092725
	Std	23.321267	34.76318932	59.42568736	54.95447158	56.28049001	38.30256467
	Best	3736.584896	5242.218307	5896.654083	4882.938027	5136.104753	5894.744809
	Worst	6637.059685	6420.08449	6606.096812	6675.235841	6768.271523	6706.157344
D31	Mean	4133.73861	5658.97124	6215.426144	5440.848598	5600.169046	6411.356336
	Std	109.343697	121.1404795	172.050326	210.6444026	213.5167965	201.3093209
	Best	2715.302689	2953.63615	3290.011686	2800.375925	2876.078555	3309.472801
	Worst	3718.291098	3840.375256	3939.087978	3952.609942	3959.489207	3995.872968
Aggregation	Mean	2789.291202	3158.484101	3672.354272	3080.247639	3112.108684	3731.786921
	Std	2.53496107	89.73403431	165.7279226	146.4400151	159.5026523	183.3215657
	Best	1060.674781	1150.328041	1279.985246	1104.072942	1120.609246	1361.339487
	Worst	1541.948974	1575.296587	1604.72384	1664.861515	1654.681553	1678.228393
Compound	Mean	1094.9423	1248.529445	1423.281446	1246.770747	1273.87772	1493.276887
	Std	13.2355642	35.63566319	79.71609294	66.55744532	71.96053364	86.13103684
	Best	1424.899542	1427.872936	1492.322506	1425.176917	1429.842419	1553.128473
	Worst	1723.311224	1676.139045	1901.140798	1857.587734	1897.234909	1893.710862
Pathbased	Mean	1430.903602	1447.009762	1683.491592	1497.152685	1477.009539	1703.054894
	Std	1.6570813	7.767526694	109.5463984	44.80756305	38.18020827	83.51041425
	Best	1807.54755	1807.510795	1832.06375	1807.595765	1808.281132	1896.181926
	Worst	2015.011175	1926.563714	2163.452999	2094.070221	2107.31257	2149.720749
Spiral	Mean	1810.02073	1809.074549	1963.454005	1820.774656	1824.186315	1996.155056
	Std	2.168093216	0.663986887	70.079482	10.71573663	17.47221358	73.8703224

**Table 4:** Comparison of objective values of MFO, BHA, MVO, HHO, GWO, k-means algorithms.

Dataset	Criteria	MFO	BHA	MVO	HHO	GWO	K-Means
	Best	254.5686207	344.1858768	427.2765574	302.6048772	360.4325397	482.794362
	Worst	607.015981	579.4491593	657.6790272	653.2463069	682.8121634	668.037993
Glass	Mean	286.3971108	394.6702904	563.6985645	375.0501591	441.7389961	592.7121853
	Std	8.5864965	14.97574454	37.57437297	29.06851845	44.90562621	50.8694328
	Best	96.6566922	102.1609776	141.6280996	105.4454434	91.06876813	155.9380716
	Worst	187.7141075	196.0131392	231.7066358	220.9449828	186.6739426	215.8188002
Iris	Mean	99.54558066	111.6727822	177.7656738	128.8472893	104.0780971	189.2905571
	Std	0.04642567	2.418165686	18.17189821	9.40506885	9.580750806	19.36588562
	Best	6176852.759	6877262.007	9811505.667	7416306.523	7788077.075	10335482.5
	Worst	10526429.84	9127679.781	11418117.43	11754852.48	11731706.85	11731057.25
Wine	Mean	6569678.631	7404560.759	10694275.29	8018085.743	8206163.788	10942626.63
	Std	103291.3436	116859.092	411754.8747	253702.4043	229142.0161	351269.2404
	Best	297.404773	399.60419	472.7558453	344.6453467	368.171845	528.3446203
	Worst	642.528356	627.3754381	772.8618998	757.8158265	730.7458876	753.5334223
Yeast	Mean	346.0571754	421.1546863	577.9115552	380.0538835	414.0104515	634.6032704
	Std	1.325687567	4.639095791	53.82131146	13.81732081	35.1425062	55.9518587

**Table 5:** Average ranking of MFO, BHA, MVO, HHO, GWO, k-means algorithms based on mean of objective values.

	MFO	BHA	MVO	HHO	GWO	K-Means
Shape Dataset	1.375	2.5	4.875	3	3.25	6
UCI Dataset	1	3	5	2.75	3.25	6

**Table 6:** Statistical results based on mean of objective Values for shape datasets.

Test Name	Statistical Value	*p*-Value	Hypothesis
Iman-Davenport	27.69026	<0.00001	Rejected
Friedman	31.92857	<0.00001	Rejected

**Table 7:** Statistical results based on mean of objective values for UCI datasets.

Test Name	Statistical Value	*p*-Value	Hypothesis
Iman-Davenport	24.99996	<0.00001	Rejected
Friedman	17.85714	0.003131	Rejected

**Table 8:** Holm’s test statistical results based on mean of objective values for Shape datasets.

i	Algorithms	Statistical Value	*p*-Value	α/i	Hypothesis
5	K-Means	4.94433	<0.00001	0.01	Rejected
4	MVO	3.74165	0.000183	0.0125	Rejected
3	GWO	2.00446	0.045027	0.0167	Not Rejected
2	HHO	1.73719	0.08237	0.025	Not Rejected
1	BHA	1.20267	0.22913	0.05	Not Rejected

**Table 9:** Holm’s test statistical results based on mean of objective values for UCI datasets.

i	Algorithms	Statistical Value	*p*-Value	α/i	Hypothesis
5	K-Means	3.77964	0.000157	0.01	Rejected
4	MVO	3.02371	0.002497	0.0125	Rejected
3	GWO	1.70084	0.088981	0.0167	Not Rejected
2	BHA	1.51186	0.130585	0.025	Not Rejected
1	HHO	1.32287	0.185902	0.05	Not Rejected

**Table 10:** The best centroids for D31 obtained by proposed approach.

Sr No.	F1	F2
C1	20.74266809	27.59365568
C2	25.50196489	24.19312765
C3	11.57011301	8.50840516
C4	25.82211536	26.17793719
C5	27.37201232	10.57384902
C6	22.08486665	5.496210514
C7	23.58523731	8.888237338
C8	22.37594806	11.79535569
C9	4.83205804	26.81225277
C10	27.50193421	17.28098473
C11	15.01686978	27.19744896
C12	6.353870768	16.21830889
C13	16.35650612	9.106767944
C14	9.968810869	23.65566343
C15	9.153853041	14.9149635
C16	23.13295757	16.05797592
C17	8.101549272	10.37341231
C18	20.47807037	18.998876
C19	4.965093478	20.47535923
C20	26.53577694	17.86530094
C21	26.03937471	14.99664186
C22	25.47861108	6.28135661
C23	12.82474767	19.1136306
C24	15.19151476	22.86896706
C25	17.80680556	12.9098126
C26	19.90521872	23.37912391
C27	17.72660498	25.58120323
C28	11.71645567	14.69915113
C29	4.624749983	10.32233599
C30	27.65379495	21.47346273
C31	15.7736913	21.06158524

**Table 11:** The best centroids for R15 obtained by proposed approach.

Sr No.	F1	F2
C1	4.189631608	12.80375838
C2	14.09450165	5.001272186
C3	8.337048918	9.062858908
C4	4.101436934	7.52179159
C5	13.97254731	14.93207276
C6	12.79155218	8.05529297
C7	8.230614736	10.92315677
C8	16.41253705	9.985521142
C9	8.646224944	16.24662551
C10	11.02097643	11.58322744
C11	9.551563967	12.06489806
C12	11.92041063	9.712070237
C13	9.967326937	10.10242535
C14	9.645716964	7.980621354
C15	8.663770617	3.772581562

**Table 12:** The best centroids for Jain obtained by proposed approach.

Sr No.	F1	F2
C1	17.03102423	15.16831711
C2	32.58459725	7.124899903

**Table 13:** The best centroids for Flame obtained by proposed approach.

Sr No.	F1	F2
C1	7.206597929	24.16493517
C2	7.301802789	17.84894502

**Table 14:** The best centroids for Aggregation obtained by proposed approach.

Sr No.	F1	F2
C1	21.42567886	22.85728939
C2	7.716573617	8.772216185
C3	32.40196366	22.05208852
C4	33.15470428	8.782254392
C5	8.938930788	22.91640128
C6	14.65416199	7.059473024
C7	20.82265142	7.249080316

**Table 15:** The best centroids for Compound obtained by proposed approach.

Sr No.	F1	F2
C1	18.77723869	18.83342046
C2	32.64318475	16.28179213
C3	37.48781021	17.33548448
C4	10.65769689	19.33852537
C5	18.67265227	9.510696233
C6	12.61754072	9.616177793

**Table 16:** The best centroids for Pathbased obtained by proposed approach.

Sr No.	F1	F2
C1	18.82903757	30.45142379
C2	11.48394236	15.73097
C3	26.16808047	16.08878767

**Table 17:** The best centroids for Spiral obtained by proposed approach.

Sr No.	F1	F2
C1	22.64471503	22.66591643
C2	11.172831	16.53101706
C3	22.08495457	10.76472807

**Table 18:** The best centroids for Glass obtained by proposed approach.

Sr No.	F1	F2	F3	F4	F5	F6	F7	F8	F9
C1	1.531719668	13.06173613	3.510979859	1.394173337	72.84637382	0.162494133	8.41076102	0.025666476	0.007523229
C2	1.52797292	12.80840956	0.246399681	1.609315064	73.83969663	0.245748967	11.78973298	0.462253331	0.257117154
C3	1.52040748	13.35918127	0.219397152	2.308129393	70.18963569	6.207528249	6.479935975	0.152869685	0.03330514
C4	1.533244544	13.8560578	3.047071044	1.202271091	70.60025867	3.494911842	7.093112782	0.306091421	0.059719952
C5	1.512538966	13.84305467	2.912665802	0.875799374	72.00128777	0.047687008	9.335062282	0.08408769	0.032376928
C6	1.5112	14.43925402	0.008206	2.085299146	73.35680382	0.457194235	8.521081118	1.11995061	0.005501446
C7	1.513266442	12.92439889	2.072428469	0.29	72.17879752	0.585345503	9.906258882	0.045962136	0.026599321

**Table 19:** The best centroids for Iris obtained by proposed approach.

Sr No.	F1	F2	F3	F4
C1	5.01229979	3.40333071	1.471677299	0.235472045
C2	6.732802141	3.067395056	5.623784792	2.106790702
C3	5.934098654	2.797688794	4.417324546	1.41492155

**Table 20:** The best centroids for Wine obtained by proposed approach.

Sr No.	C1	C2	C3
F1	39,986.76285	43,544.94447	20,030.90947
F2	28,115.519	15,541.15111	13,971.82923
F3	45,777.07237	35,143.40404	31,390.39269
F4	28,154.45346	21,489.64815	33,270.71013
F5	21,025.39322	25,555.71232	19,697.48292
F6	16,405.36654	46,363.61618	27,124.14348
F7	16,940.6724	35,341.31586	22,796.4139
F8	37,050.85547	18,628.032	29,821.29914
F9	19,508.26413	31,543.63104	24,125.44547
F10	32,628.78338	23,408.28137	15,972.93531
F11	10,576.51405	31,095.9809	29,682.27216
F12	14,613.20707	45,340.52047	34,586.81203
F13	16,507.21954	37,817.65194	10,303.62763

**Table 21:** The best centroids for Yeast obtained by proposed approach.

Sr No.	F1	F2	F3	F4	F5	F6	F7	F8
C1	0.757337919	0.142268616	0.827461959	0.001450393	0.527740868	0.771304322	0.630304293	0.383528402
C2	0.781314193	0.71779793	0.419456881	0.377730495	0.560817461	0.015464843	0.511164619	0.170202938
C3	0.496325357	0.491261885	0.499102561	0.234178288	0.500528038	0	0.504793757	0.25014915
C4	0.131413932	0.34929326	0.393064657	0.841116915	0.704378018	0.212988264	0.518108105	0.444142521
C5	0.957824847	0.549712612	0.456841891	0.964282073	0.540272009	0.393364094	0.288371843	0.448492104
C6	0.147129502	0.724553473	0.474471507	0.175108699	0.571043788	0.746038613	0.534680335	0.185879771
C7	0.430257651	0.47424918	0.534401249	0.225056925	0.500017048	0	0.478658653	0.655020513
C8	0.371314927	0.342973839	0.518372939	0.135213842	0.521916885	0.016021841	0.545742633	0.275096267
C9	0.292646344	0.132663231	0.270567884	0.035813911	0.505437432	0.366876625	0.08142005	0.187474957
C10	0.411909662	0.491403883	0.541493781	0.519251596	0.546134059	0.000446405	0.4844054	0.113730494

## Data Availability

Not applicable.
